# Heterogeneity of treatment effect? What baseline prognostic factors tell us about immunotherapy benefit

**DOI:** 10.1093/jncics/pkag031

**Published:** 2026-04-28

**Authors:** Chee Khoon Lee, R John Simes

**Affiliations:** NHMRC Clinical Trials Centre, University of Sydney, Camperdown, NSW, Australia; NHMRC Clinical Trials Centre, University of Sydney, Camperdown, NSW, Australia

Immune checkpoint inhibitors (ICIs) have transformed the treatment landscape across multiple advanced cancers. Yet despite their profound impact, clinical outcomes remain highly variable. While some patients achieve durable benefit, many experience limited or no response. Understanding this variability is a central challenge in oncology and underpins growing interest in heterogeneity of treatment effect (HTE).

In oncology trials, subgroup analyses are routinely performed to assess whether baseline patient characteristics modify treatment effects, with the aim of identifying meaningful differences in benefit that could inform clinical practice. However, conventional one-variable-at-a-time subgroup analyses are widely recognized as problematic: they are underpowered, susceptible to multiplicity, and often difficult to interpret clinically. Patients do not present with isolated characteristics, and analyzing interactions one factor at a time may risk obscuring clinically meaningful patterns. Outside oncology, the concept that baseline risk modifies treatment benefit is well-established, particularly in the cardiovascular setting, as the “average” effect observed in a randomized controlled trial (RCT) may not reflect the benefit experienced by a typical patient.^1^^,^^2^ To move beyond one-variable-at-a-time subgroup analyses, multivariable risk-modelling approaches integrate multiple prognostic factors to quantify baseline risk. Within this framework,^3^ studies evaluating more than 30 trials have shown that patients at higher baseline risk usually experience similar relative treatment effects, but derive greater absolute benefit from the effective therapies.^4^^,^^5^ Exploring variation in relative treatment effects as a function of baseline risk has therefore been recommended when there is a large overall relative treatment effect,^6^ as it can help refine estimates of absolute benefit for clinical decision-making. This approach, however, is usually not recommended when no overall treatment effect has been demonstrated.

Whether such a paradigm can be translated meaningfully into oncology, particularly in the context of ICI, remains uncertain. In this issue, Li et al.^7^ address this question in a post hoc analysis applying a risk-modelling framework to individual participant data from 10 phase III RCTs of atezolizumab across several advanced cancers. Their central aim is to determine whether risk scores derived from multivariable prognostic modelling could function as effect modifiers for atezolizumab.

Across 9 of the 10 RCTs examined, including all 6 studies supporting regulatory approval of atezolizumab, Li et al. find no convincing evidence that baseline prognosis modifies relative treatment benefit. Despite advanced modelling techniques, continuous interaction testing, and multiple sensitivity analyses, treatment effects appear broadly homogeneous across the prognostic spectrum. This largely negative finding is itself important. It suggests that, within the confines of RCT populations, baseline prognosis as captured by clinical and laboratory variables does not generally function as a predictive factor for ICI benefit on the relative scale. This mirrors experience in cardiovascular RCTs, where most studies show little or no HTE on a relative scale but only on an absolute scale.

Several plausible explanations may account for the lack of detectable HTE observed by Li et al. These RCTs enrolled highly selected and relatively homogeneous populations, with strict eligibility criteria related to performance status, organ function, and disease stage. Consequently, the range and dispersion of baseline risk are artificially compressed, limiting both the power and plausibility of detecting treatment-risk interactions. Among the baseline variables examined, tumor PD-L1 expression is the most widely recognized as a potentially predictive biomarker for ICI, yet its predictive performance is inconsistent across cancer types.^8^ In RCTs evaluating the addition of atezolizumab to chemotherapy, there is some evidence of greater relative benefit in non-small cell lung tumor with higher PD-L1 expression,^9^ but such expression is also linked to better prognosis in these patients receiving chemotherapy alone.^10^ More broadly, however, ICI outcomes are governed by complex biological processes, such as tumor-immune interactions and micro-environmental dynamics,^11^ that are poorly captured by baseline variables and remain only partially understood.

For clinicians, this finding may be both reassuring and unsurprising in the absence of strong biological effect modifiers linked to baseline prognosis. The absence of detectable HTE suggests that the relative treatment effects observed in these pivotal atezolizumab RCTs can, at least within the confines of trial populations, be reasonably applied across a broad range of patients. This may simplify treatment decisions and reduce the temptation to over-interpret subgroup analyses. However, this apparent homogeneity of relative effect reflects the highly selected nature of trial populations and does not preclude clinically meaningful heterogeneity emerging in broader real-world settings, where variation in disease subtype, tumor biology, or treatment sequencing—rather than simply worse prognosis—may meaningfully influence ICI benefit.

Against this largely null background, one trial stands apart. In IMvigor211, a phase III RCT comparing atezolizumab with chemotherapy in platinum-refractory metastatic urothelial carcinoma, Li et al. identify apparently strong evidence of HTE. However, caution is warranted when interpreting this finding. IMvigor211 is a negative trial by its primary analysis, with no statistically significant overall survival benefit.^12^ In such settings, statistically significant interaction effects warrant heightened skepticism. Without an overall treatment effect, interaction signals are more vulnerable to chance findings, modeling artefacts, or unmeasured confounding, and recommendations to explore HTE by baseline risk are less well supported.^6^

An important aspect of this analysis is the need to distinguish between trial designs when evaluating HTE. Most of the included RCTs assessed the addition of atezolizumab to an established systemic treatment backbone, whereas others evaluated substitution of atezolizumab for standard-of-care chemotherapy or targeted therapy. In add-on RCTs, any interaction between baseline risk and treatment effect can reasonably be attributed to the incremental effect of atezolizumab. In substitution RCTs, however, observed interactions may plausibly arise from variation in the performance of either arm—or both—rather than from differential efficacy of atezolizumab alone. For clinicians, this distinction is important. If baseline prognosis appears to modify benefit in an add-on trial, this could support selectively adding atezolizumab to an established treatment backbone in certain risk groups. By contrast, a similar finding in a substitution trial could simply indicate that standard-of-care therapy performs better than atezolizumab in specific prognostic strata, rather than that atezolizumab is less effective per se. Without explicit differentiation between these trial designs, treatment-risk interactions become difficult to interpret causally and risk being misread as evidence of differential ICI efficacy when they may instead reflect variation in the comparator regimen.

Given that risk-modeling approaches seldom identify HTE in oncology RCTs, an important question is whether assessing HTE remains a useful exercise. The answer is yes—but not primarily as a tool for discovering predictive subgroups. Even when relative treatment effects are constant, baseline risk influences absolute benefit. A modest hazard ratio may translate into a larger absolute survival gain in a high-risk than a low-risk population. Conversely, high-risk individuals may still experience only a modest extension in expected survival duration, as their underlying prognosis remains poor. Prognostic models, therefore, remain valuable for contextualizing treatment effects, informing shared decision-making, and weighing benefits against toxicity, cost, and patient preferences. However, the utility of this approach depends critically on the choice and validity of baseline variables. Risk models must be rigorously validated and reflect key, disease-specific prognostic factors for the diseases being studied.^13^ Reliance on generic prognostic factors applied across heterogeneous advanced cancer populations, as in the work of Li et al., risks diluting clinically meaningful prognostic stratification.

Taken together, the evidence presented by Li et al. suggests that routine prognostic subgrouping is unlikely to yield clinically actionable insights in most ICI RCTs of advanced cancers, particularly when applied indiscriminately. Instead, a more nuanced approach is warranted: prioritizing add-on trial designs when assessing HTE, pre-specifying prognostic interaction analyses where biologically plausible, and using prognostic models primarily to inform absolute benefit.

In conclusion, Li et al. provide a comprehensive evaluation of prognosis-based HTE in atezolizumab RCTs across multiple advanced cancers. Their analysis demonstrates that, as in cardiovascular medicine, baseline prognosis usually does not modify relative treatment benefit. Nevertheless, baseline risk remains critical for guiding decision-making by determining the magnitude of absolute benefit and the balance of benefit and harm. This work advances the discussion beyond the simplistic one-variable-at-a-time subgroup analyses toward more rigorous multivariable risk-modelling approaches for evaluating HTE. Continued efforts to discover predictive biomarkers linked to ICI mechanisms will remain essential. However, the key lesson here is that prognosis-based risk modelling is often most valuable not for detecting HTE, but for helping estimate differences in absolute benefit to guide decision making in an era increasingly focused on personalized care.

## Data Availability

No new data were generated or analyzed for this editorial.

## References

[1] Rothwell PM. Can overall results of clinical trials be applied to all patients? Lancet. 1995;345:1616-1619. 7783541 10.1016/s0140-6736(95)90120-5

[2] Kent DM , Steyerberg E, van Klaveren D. Personalized evidence based medicine: predictive approaches to heterogeneous treatment effects. BMJ. 2018;363:k4245. 30530757 10.1136/bmj.k4245 PMC6889830

[3] Kent DM , Rothwell PM, Ioannidis JPA, et al Assessing and reporting heterogeneity in treatment effects in clinical trials: a proposal. Trials. 2010;11:85. 20704705 10.1186/1745-6215-11-85 PMC2928211

[4] Kent DM , Nelson J, Dahabreh IJ, et al Risk and treatment effect heterogeneity: re-analysis of individual participant data from 32 large clinical trials. Int J Epidemiol. 2016;45:2075-2088. 27375287 10.1093/ije/dyw118 PMC5841614

[5] Sussman JB , Kent DM, Nelson JP, Hayward RA. Improving diabetes prevention with benefit based tailored treatment: risk based reanalysis of Diabetes Prevention Program. BMJ 2015;350:h454. 25697494 10.1136/bmj.h454 PMC4353279

[6] Kent DM , Paulus JK, van Klaveren D, et al The Predictive Approaches to Treatment effect Heterogeneity (PATH) Statement. Ann Intern Med. 2020;172:35-45. 31711134 10.7326/M18-3667 PMC7531587

[7] Li LX , Shahnam A, Kichenadasse G, et al Heterogeneous treatment effect of immune checkpoint inhibitors by pre-treatment prognosis in randomised controlled trials. JNCI Cancer Spectrum. 2026. 10.1093/jncics/pkaf127 PMC13126119 41501995

[8] Davis AA , Patel VG. The role of PD-L1 expression as a predictive biomarker: an analysis of all US Food and Drug Administration (FDA) approvals of immune checkpoint inhibitors. J ImmunoTher Cancer. 2019;7:278. 31655605 10.1186/s40425-019-0768-9 PMC6815032

[9] Fitri NK , Nainggolan BWM, Firsty NN, et al The addition of atezolizumab to chemotherapy in non-small cell lung cancer: a trial-based review and meta-analysis. World J Oncol. 2024;15:72-80. 38274722 10.14740/wjon1701 PMC10807920

[10] Woodford R , Zhou D, Lord SJ, et al PD-L1 expression as a prognostic marker in patients treated with chemotherapy for metastatic non-small-cell lung cancer. Future Oncol. 2022;18:1793-1799. 35156837 10.2217/fon-2021-1184

[11] Havel JJ , Chowell D, Chan TA. The evolving landscape of biomarkers for checkpoint inhibitor immunotherapy. Nat Rev Cancer. 2019;19:133-150. 30755690 10.1038/s41568-019-0116-x PMC6705396

[12] Powles T , Durán I, van der Heijden MS, et al Atezolizumab versus chemotherapy in patients with platinum-treated locally advanced or metastatic urothelial carcinoma (IMvigor211): a multicentre, open-label, phase 3 randomised controlled trial. The Lancet. 2018;391:748-757. 10.1016/S0140-6736(17)33297-X 29268948

[13] Steyerberg EW , Moons KGM, van der Windt DA, et al; PROGRESS Group. Prognosis Research Strategy (PROGRESS) 3: prognostic model research. PLoS Med. 2013;10:e1001381. 23393430 10.1371/journal.pmed.1001381 PMC3564751

